# Supplementary material for: Proteotranscriptomic Profiling of the Toxic Mucus of Kulikovia alborostrata (Pilidiophora, Nemertea)
Source: Toxins (Basel). 2024 Dec 26;17(1):5. doi: 10.3390/toxins17010005 (PMC11768458; doi:10.3390/toxins17010005)
Supplement: Supplementary file 1 [file toxins-17-00005-s001.zip › Supplementary File S4.zip!/ORF 001200 LINALB/1362d454f90accc2/hit_report.pdf]

Phyre2

Email	vas9i-kz@mail.ru
Description	ORF_001200_LINALB
Date	Fri Apr 7 03:17:43 BST 2023
Unique Job ID	1362d454f90accc2

Detailed template information

#	Template	Alignment Coverage	3D Model	Confidence	% i.d.	Template Information
1	c2f2jA_	Alignment	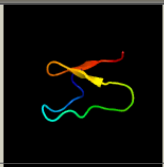	81.2	61	**PDB header:** antimicrobial protein<br>**Chain:** A: **PDB Molecule:** kalata-b1;<br>**PDBTitle:** solution structure of [w19k, p20n, v21k]-kalata b1<br>**PDB Entry:** PDBe RCSB PDBj
2	d1n1ua_	Alignment	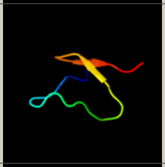	67.2	61	**Fold:** Knottins (small inhibitors, toxins, lectins)<br>**Superfamily:** Cyclotides<br>**Family:** Kalata B1<br>**PDB entry:** PDBe RCSB PDBj
3	c1n1uA_	Alignment	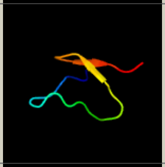	67.2	61	**PDB header:** antibiotic<br>**Chain:** A: **PDB Molecule:** kalata b1;<br>**PDBTitle:** nmr structure of [ala1,15]kalata b1<br>**PDB Entry:** PDBe RCSB PDBj
4	c7k7xA_	Alignment	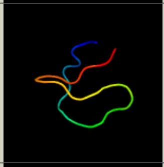	62.5	61	**PDB header:** immunosuppressant<br>**Chain:** A: **PDB Molecule:** cyclotide pase a;<br>**PDBTitle:** solution structure of the cyclotide pase a<br>**PDB Entry:** PDBe RCSB PDBj
5	c4ttoA_	Alignment	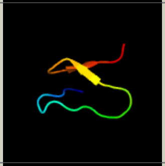	60.5	61	**PDB header:** plant protein<br>**Chain:** A: **PDB Molecule:** kalata-b1;<br>**PDBTitle:** quasi-racemic structure of [v25a] kalata b1<br>**PDB Entry:** PDBe RCSB PDBj
6	c4ttmA_	Alignment	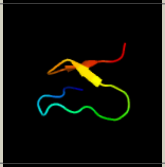	59.2	61	**PDB header:** plant protein<br>**Chain:** A: **PDB Molecule:** kalata-b1;<br>**PDBTitle:** racemic structure of kalata b1 (kb1)<br>**PDB Entry:** PDBe RCSB PDBj
7	d1nb1a_	Alignment	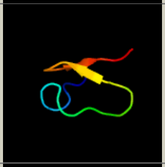	59.0	61	**Fold:** Knottins (small inhibitors, toxins, lectins)<br>**Superfamily:** Cyclotides<br>**Family:** Kalata B1<br>**PDB entry:** PDBe RCSB PDBj
8	c1nb1A_	Alignment	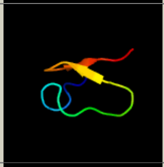	59.0	61	**PDB header:** antibiotic<br>**Chain:** A: **PDB Molecule:** kalata b1;<br>**PDBTitle:** high resolution solution structure of kalata b1<br>**PDB Entry:** PDBe RCSB PDBj
9	c2khbA_	Alignment	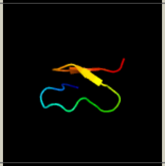	58.9	61	**PDB header:** antimicrobial protein<br>**Chain:** A: **PDB Molecule:** kalata-b1;<br>**PDBTitle:** solution structure of linear kalata b1 (loop 6)<br>**PDB Entry:** PDBe RCSB PDBj
10	c7lhcA_	Alignment	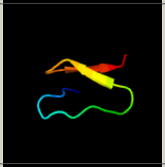	58.0	61	**PDB header:** plant protein<br>**Chain:** A: **PDB Molecule:** kalata-b1;<br>**PDBTitle:** nmr solution structure of [t20k]kalata b1<br>**PDB Entry:** PDBe RCSB PDBj
11	c2mn1A_	Alignment	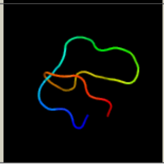	37.7	61	**PDB header:** unknown function<br>**Chain:** A: **PDB Molecule:** kalata b1[w23ww];<br>**PDBTitle:** solution structure of kalata b1[w23ww]<br>**PDB Entry:** PDBe RCSB PDBj

12	c4ttnA_	Alignment	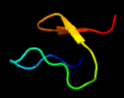	57.8	57	**PDB header:**plant protein<br>**Chain:** A: **PDB Molecule:**kalata-b1;<br>**PDBTitle:** quasi-racemic structure of [g6a]kalata b1<br>**PDB Entry:** PDBe RCSB PDBj
13	d1pt4a_	Alignment	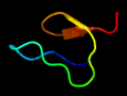	57.5	57	**Fold:** Knottins (small inhibitors, toxins, lectins)<br>**Superfamily:** Cyclotides<br>**Family:** Kalata B1<br>**PDB entry:** PDBe RCSB PDBj
14	c2kukA_	Alignment	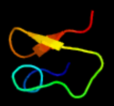	39.3	57	**PDB header:**antiviral protein<br>**Chain:** A: **PDB Molecule:**leaf cyclotide 2;<br>**PDBTitle:** solution structure of vhl-2<br>**PDB Entry:** PDBe RCSB PDBj
15	c7rfaA_	Alignment	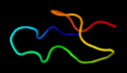	35.9	59	**PDB header:**biosynthetic protein<br>**Chain:** A: **PDB Molecule:**kalata-b4;<br>**PDBTitle:** nmr solution structure of linear [t20k]kalatab1<br>**PDB Entry:** PDBe RCSB PDBj
16	c1jjzA_	Alignment	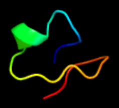	35.3	59	**PDB header:**plant protein<br>**Chain:** A: **PDB Molecule:**kalata-b4;<br>**PDBTitle:** refined structure and disulfide pairing of the kalata b1 peptide<br>**PDB Entry:** PDBe RCSB PDBj
17	c1k48A_	Alignment	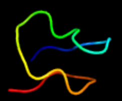	33.4	59	**PDB header:**plant protein<br>**Chain:** A: **PDB Molecule:**kalata b1;<br>**PDBTitle:** refined structure and disulfide pairing of the kalata b1 peptide<br>**PDB Entry:** PDBe RCSB PDBj
18	c2f2iA_	Alignment	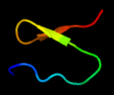	50.1	63	**PDB header:**antimicrobial protein<br>**Chain:** A: **PDB Molecule:**kalata-b1;<br>**PDBTitle:** solution structure of [p20d,v21k]-kalata b1<br>**PDB Entry:** PDBe RCSB PDBj
19	c2jwmA_	Alignment	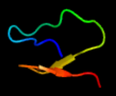	30.0	46	**PDB header:**plant protein<br>**Chain:** A: **PDB Molecule:**kalata-b7;<br>**PDBTitle:** nmr spatial srtucture of ternary complex kalata b7/mn2+/dpc micelle<br>**PDB Entry:** PDBe RCSB PDBj
20	c3e4hA_	Alignment	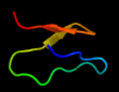	15.6	46	**PDB header:**plant protein<br>**Chain:** A: **PDB Molecule:**varv peptide f;<br>**PDBTitle:** crystal structure of the cyclotide varv f<br>**PDB Entry:** PDBe RCSB PDBj
21	c2m9oA_	Alignment	not modelled	15.4	46	**PDB header:**plant protein<br>**Chain:** A: **PDB Molecule:**kalata-b7;<br>**PDBTitle:** solution structure of kalata b7<br>**PDB Entry:** PDBe RCSB PDBj
